# Smart Textile Flexible MnCo_2_O_4_ Electrodes: Urea Surface Modification for Improved Electrochemical Functionality

**DOI:** 10.3390/ma17081866

**Published:** 2024-04-18

**Authors:** Manesh A. Yewale, Aviraj M. Teli, Sonali A. Beknalkar, Vineet Kumar, Dong-Kil Shin

**Affiliations:** 1 School of Mechanical Engineering, Yeungnam University, Gyeongsan 38541, Republic of Korea; maneshphd@gmail.com (M.A.Y.); vineetfri@gmail.com (V.K.); 2 Division of Electronics and Electrical Engineering, Dongguk University-Seoul, Seoul 04620, Republic of Korea; avteli.teli@gmail.com (A.M.T.); sonaliabeknalkar@gmail.com (S.A.B.)

**Keywords:** MnCo_2_O_4_ nanoparticles, supercapacitor, TEM, hydrothermal, asymmetric supercapacitor

## Abstract

Surface microstructure modification of metal oxides also improves the electrochemical performance of metal oxide nanoparticles. The present investigation demonstrates how varying the urea molar content during the hydrothermal process altered the surfaces of MnCo_2_O_4_ nanoparticles. Successive increases of 0.1 M in urea concentration transformed the surface shape of MnCo_2_O_4_ nanoparticles from flower-like to sheet-like microstructures. Excellent electrochemical performance of MnCo_2_O_4_ nanoparticles was demonstrated in an aqueous 1 M KOH electrolyte. The improved MnCo_2_O_4_ nanoparticles have been employed to develop an asymmetric supercapacitor (ASC). The ASC device exhibits an energy density of 13 Wh/kg at a power density of 553 W/kg and a specific capacitance of 29 F g^−1^ at a current density of 4 mA/cm^2^. The MnCo_2_O_4_ nanoparticle electrode demonstrates remarkable electrocatalytic activity in both HER and OER. The MnCo_2_O_4_ electrode shows overpotential for HER and OER at 356 mV and 1.46 V, respectively. The Tafel slopes for HER and OER of the MnCo_2_O_4_ electrode are 356 mV/dec and 187 mV/dec, respectively.

## 1. Introduction

The persistent consumption of petroleum and natural gas, propelled by an expanding global population, leads to natural resource deterioration and the emergence of serious energy shortages. Renewable energy sources paired with more effective technology for storing energy, represent an avenue for helping reduce pollution and improving energy challenges [1,2,3,4,5,6,7]. The accessibility to renewable energy sources, which are free of pollution, is essential for the development of the vehicle industry and human civilization as a whole. Electrocatalysis of the hydrogen evolution reaction (HER) and the oxygen evolution reaction (OER) are green energy alternatives that might be considered. Preparing nanoparticles for use in electrocatalysis can accomplish this. In order to solve future energy problems, HER use is crucial. As a result, hydrogen is going to be an essential component of sustainable energy systems in the future [8,9,10]. Electronic devices, whether powered by batteries or directly by sources such as electric cars, have seen enormous expansion, altering the landscape of fossil fuel-dependent gadgets [11,12,13,14]. Upcoming needs for energy indicate the necessity of high-energy and high-power sources. Nonetheless, while batteries are frequently used, they have a lower power density than supercapacitors. Supercapacitors gain more attention than batteries due to their inherent properties of rapid charge times, extended life, and flexibility. The flexibility of flexible supercapacitors, along with their simplicity of setup, makes them especially appropriate for a wide range of applications. The operational voltage window of hybrid solid-state supercapacitors has been expanded, resulting in higher energy density [15,16]. Supercapacitors have been separated into two varieties based on their charge storage mechanisms: pseudocapacitors and electric double-layer capacitors (EDLC). Metal oxides and chalcogenides primarily demonstrate pseudocapacitor operation; however, carbon-based materials exhibit EDLC behavior. Transition metal oxides, including monometallic and bimetallic, have been getting an abundance of interest recently. Bimetallic oxides having spinel structures, such as AB_2_O_4_ (B=Co; A=Cu, Ni, Zn, Mn, etc.), have attracted substantial study focus for energy storage applications such as supercapacitors and electrolysis [17,18,19,20]. The synergy of two metals in the bimetallic oxides improves specific capacity, and the presence of numerous oxidation states accelerates faradic redox processes, which increases storage capacity even more [21,22,23,24,25]. Cobalt cation substitutes in bimetallic oxides alongside elements such as Zn, Ni, Cu, and Mn, offer benefits such as lower expenses, better conductivity of electricity, higher redox sites, and ecological friendliness. As a result of their greater valence state over Zn or Cu, manganese-based metal oxides have attracted quite a bit of attention [25,26]. MnCo_2_O_4_ exceeds monometal oxides such as MnO_2_ and Co_3_O_4_ in electrochemical performance. Several methods to manufacture MnCo_2_O_4_ nanoparticles with various microstructures (flowers, rods, and wires) in aqueous and non-aqueous solvents on favorable or flexible carbon cloth substrates have been reported. The electrochemical performance varies depending on the synthesis method, the surface microstructure, the solvent, and the substrate.

In the current article, we explore surface microstructure tailoring in the hydrothermal process by altering urea content. Hydrothermal approaches were used to synthesize MnCo_2_O_4_ nanoparticles with urea concentrations that varied from 0.2 M to 0.5 M. Electrochemical performance was studied using electrodes made from flexible carbon fabric. Electrodes developed with a 0.3 M molar urea concentration in a 2 M potassium hydroxide electrolyte displayed remarkable electrochemical performance. Surface microstructure tweaking resulted in compelling variations in electrode performance, with nanowires linking to larger active sites and boosting energy storage capacity and electrocatalytic performace of the electrode. Electrodes and devices made from 0.3 M nanoparticles exhibited remarkable specific capacitance, energy density, power density and electrocatalytic performance.

## 2. Experimental

The hydrothermal method was used to synthesize the MnCo_2_O_4_ nanoparticles. The following solutions were prepared by dissolving the following in 40 mL of deionized water: 0.1 M Mn(NO_3_)_2_·6H_2_O, 0.2 M Co(NO_3_)_2_·6H_2_O, and 0.2 M NH_2_CONH_2_. After 30 min of stirring, the solution was considered homogenous. After pouring the entire solution into a 100 mL sealed Teflon hydrothermal liner, it was put in a stainless-steel reactor. After being sealed, the reactor was placed in the oven. The hydrothermal process continued for nine hours at a constant temperature of 120 °C. The hydrothermal reactor was allowed to cool down to room temperature naturally once the reaction was finished. After the hydrothermal reaction was complete, the product was filtered through filter paper after several rinses with water and ethanol. After filtering, the precipitate was transferred in a Petri dish and left to dry overnight at 60 °C in the oven. The dried product was subsequently placed in a crucible and subjected to an annealing process at 400 °C for a duration of four hours to produce MnCo_2_O_4_ nanoparticles. The MnCo_2_O_4_ nanoparticles were named MCO-0.2 molar urea (MU) after cooling to room temperature following the annealing procedure. The MCO-0.3 MU, MCO-0.4 MU, and MCO-0.5 MU MnCo_2_O_4_ nanoparticles were synthesized using the same procedure, by tuning urea concentrations to 0.3 M, 0.4 M, and 0.5 M, respectively. All sections of the article, including characterization, findings, and explanations, utilized the same terminology. Subsequent research and analysis made use of each sample.

### 2.1. Material Characterization

The specimens underwent crystallographic examination using X-ray diffraction (XRD) with PAN analytical equipment, employing Cu-Kα radiation. The oxidation states of the surface elements were determined using X-ray photoelectron spectroscopy (XPS) with K-alpha equipment from Thermo Scientific, Oxford, UK. Surface morphology and energy dispersive spectroscopic (EDS) analysis were conducted using a HITACHI Ltd. Tokyo, Japan S-4800 field-emission scanning electron microscope (FE-SEM). Electrochemical data were acquired using a potentiostat (WonAtech, Zive SP-5, Seoul, Republic of Korea). A Tecnai F21 high-resolution transmission electron microscope (HRTEM) from FEI Company (Hillsboro, OR, USA) was used for a more comprehensive analysis of surface morphology and element mapping. 

### 2.2. Electrode Preparation 

Conducting stability tests, electrochemical impedance spectroscopy (EIS), cyclic voltammetry (CV), and galvanostatic charge-discharge (GCD) studies, the MnCo_2_O_4_ nanoparticles were electrochemically studied at the Wonatech (SP-5) workstation. The carbon cloth (CC) electrodes measuring 1 × 2 cm^2^ were used to prepare the electrodes. In NMP solvent, MnCo_2_O_4_ nanoparticles, PVDF, and carbon black were mixed in a weight percentage ratio of 80:10:10. Next, a 1 × 1 cm^2^ surface area CC was used to drop cast the prepared solution. In order to prepare the electrodes for further electrochemical investigations, they were oven-dried overnight at 60 degrees Celsius. The asymmetric supercapacitors were assembled using two electrodes: one made of optimized MnCo_2_O_4_ and the other of activated carbon (YEC-8A). Activated carbon electrodes are made in an identical way as MnCo_2_O_4_ electrodes, but instead of employing nanoparticles of MnCo_2_O_4_, activated carbon was employed.

## 3. Result and Discussion

### 3.1. X-ray Diffraction and X-ray Photoelectron Spectroscopy (XPS) Analysis

**Figure 1:**
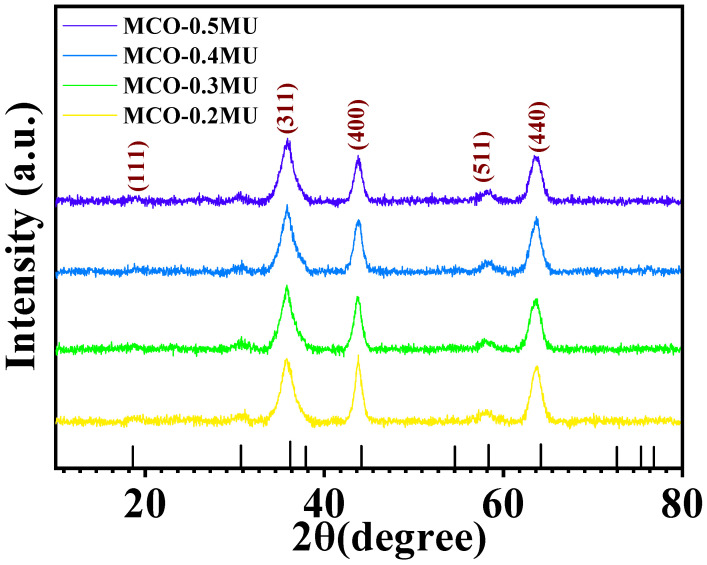
XRD spectra of MnCo_2_O_4_ nanoparticles prepared at different molar concentration of urea.

**Figure 2:**
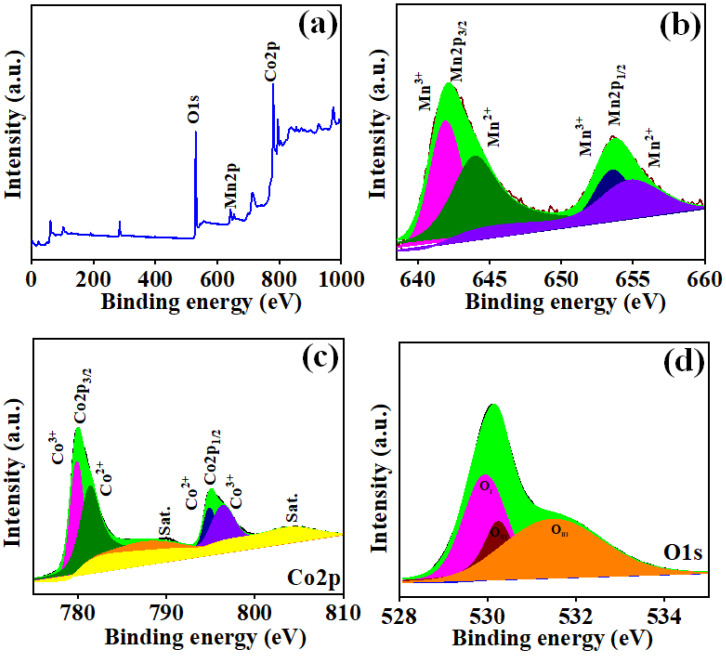
XPS spectra of MCO-0.3 MU (**a**) survey spectrum, (**b**), Mn2p, (**c**) Co2p and (**d**) O1s.

The analysis of XRD was employed to evaluate the structural phases and structure of the MnCo_2_O_4_ nanoparticles. Figure 1 demonstrates the XRD spectra of MnCo_2_O_4_ nanoparticles synthesized by various urea molar concentrations that vary between 0.2 to 0.5 M. The spectra reveal slight variations in peak intensity and sharpness. With rising urea molar concentrations, the peak intensity associated to the (400) plane diminishes. The nanoparticle XRD spectrum at 0.3 M concentration appears sharper than the other XRD spectra. Peaks can be recognized in all the XRD results at 18.77, 35.82, 43.66, 58.53, and 63.66, which correspond to (111), (311), (400), (511), and (440), respectively. These peaks are completely comparable to JCDPS card 00-001-1130, displaying the cubic phase growth of MnCo_2_O_4_ nanoparticles with lattice parameters of 8.20Å. The X-ray photoelectron spectroscopy (XPS) details were used to study the MnCo_2_O_4_ nanoparticles’ oxidation state and composition. Figure 2a displays the XPS spectra obtained from the survey scan of the MCO-0.3 MU nanoparticle. The spectra illustrate that the prepared sample incorporates oxygen, manganese, and cobalt. Additionally, Figure 2b displays the Mn2p’s high-resolution spectra. The convolution and deconvolution of the Mn2p spectra revealed two prominent peaks at 642.13 eV and 653.74 eV, respectively, which correspond to the Mn 2p_3/2_ and Mn 2p_1/2_ oxidation states. The occurrence of the Mn^3+^ state appears by the peaks at 641.90 eV and 653.58 eV, whereas the existence of the Mn^2+^ state is shown by the peaks at 644.02 eV and 654.95 eV, respectively [27]. Figure 2c displays the four-peak fitted convolution and deconvolution XPS spectra of the Co2p. It is also proposed to attribute peaks at 779.90 eV and 794.85 eV to Co^3+^, and peaks at 781.41 eV and 796.42 eV to Co^2+^. It is Co2p that appears as the satellite peak at 784.73 eV and 804.24 eV [28,29,30]. Three peaks at 529.93, 530.23, and 531.52 corresponding to Mn-O, C-O, and C=O bonds, respectively, are depicted in Figure 2d as the O1s’ high-resolution spectra [28]. The results showed considerable matching with the XRD and EDS spectra and the XPS data, indicating that MnCo_2_O_4_ nanoparticles had been formed.

### 3.2. Morphological Analysis

**Figure 3:**
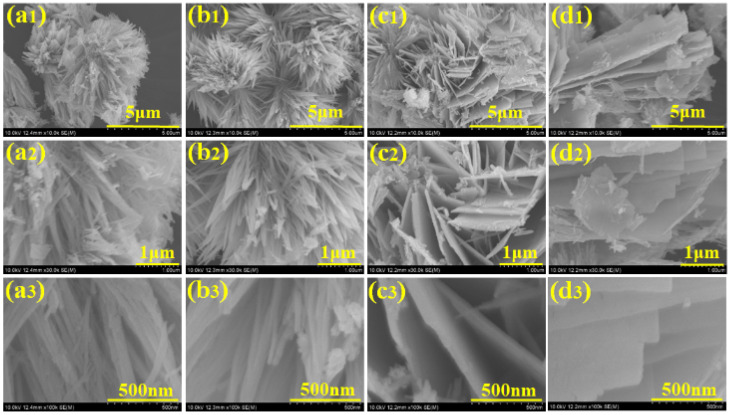
FESEM micrograph of MnCo_2_O_4_ nanoparticles at different magnifications, MCO-0.2 MU (**a1**–**a3**), MCO-0.3 MU (**b1**–**b3**), MCO-0.4 MU (**c1**–**c3**), MCO-0.5 MU (**d1**–**d3**).

**Figure 4:**
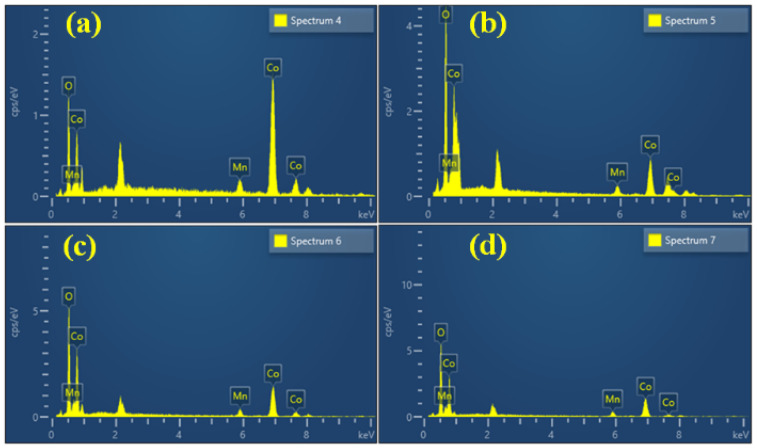
EDS spectra of MnCo_2_O_4_ nanoparticles, MCO-0.2 MU (**a)**, MCO-0.3 MU (**b**), MCO-0.4 MU (**c**), MCO-0.5 MU (**d**).

**Figure 5:**
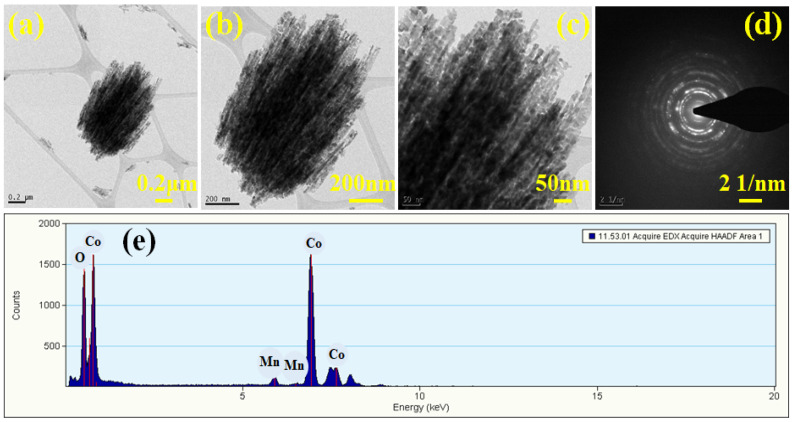
TEM micrograph at different magnifications (**a**–**c**), SAED pattern (**d**), EDS spectra (**e**) of MnCo_2_O_4_ nanoparticles (MCO-0.3 MU).

The surface morphology of nanomaterials has an important effect on their electrochemical activity. The surface microstructure of the nanoparticles was examined using field-scanning electron microscopy (FESEM). Figure 3 depicts a FESEM micrograph of MnCo_2_O_4_ nanoparticles synthesized using various urea concentrations in molar quantities and acquired at various magnifications. Figure 3(a1–a3) depicts a MnCo_2_O_4_ nanoparticle produced at a urea concentration of 0.2 M. The FESEM micrograph shows the beginnings of the growth of MnCo_2_O_4_ nanoparticles with a flower-like shape, as well as the formation of the flower’s petals. The petals of the MnCo_2_O_4_ nanoparticles join together at the flower base and begin to expand in every direction, indicating the earliest flower’s growth. A few of the petals combine jointly, providing a compact flower-like shape, and the influence on the electrode’s electrochemical performance was observed. The compactness of the petals may make the insertion and extension of ions challenging, delivering a small impediment during the charging and discharging process, resulting in limited charge storage capacity of the electrode. The surface microstructure of the nanoparticles demonstrated the tuning of the floral structure alteration as the molar concentration of urea was set to 0.3 M. The development of the nanoparticles is accelerated at 0.3 M molar concentrations of urea, and distinct petals of the flower are visible. The flower’s petals are fully opened from the free end side, allowing free space between each petal, and each petal resembles a wire-like structure, as observed in Figure 3(b1–b3). The ends of a few petals are attached from the free end together in the FESEM micrograph, and the bottom is likewise already joined with free spacing between the petals. All of these clumped petal structures combine to form a flower-like shape with open space between each petal. The effect of free space and the connecting of the tip of the petal on electrochemical performance in energy storage capacity has been identified. The free space enables effortless access for ions during charging and discharging, as revealed by a longer discharge time for the MCO-0.3 MU electrode in GCD as well as reduced series resistance and charge transfer resistance in the EIS study. At a 0.4 M urea concentration, vital modifications to the surface were detected. Figure 3(c1–c3) depicts a FESEM micrograph of MnCo_2_O_4_ nanoparticles generated at 0.4 M urea concentration, revealing their modification from a flower-like morphology to a sheet-like structure. The petals of the flower in 0.3 M urea concentration clumped together laterally and began to overlap, resulting in the sheet-like morphology shown in the FESEM micrographs. As sheet making begins, barely any top and bottom surfaces are accessible for electrolyte and electrode interaction, and the surface microstructure tailoring results in electrochemical performance. Figure 3(d1–d3) depicts a FESEM micrograph of MnCo_2_O_4_ nanoparticles with a consistent sheet-like surface shape at 0.5 M urea concentration. With urea concentration variations, the flower-like surface microstructure is entirely lost, transforming it into a sheet-like microstructure. The surface morphology adjustment also affects the electrode’s electrochemical performance. As a whole, the petal-like microstructure demonstrates a flower-like shape with free petal-like structures at 0.3 M urea concentration, exhibiting better electrochemical performance, possibly due to the open space for interaction between the electrode and electrolyte. EDS analysis was used to figure out the elemental composition of the produced nanoparticles. Figure 4a–d depicts the EDS spectra of all MnCo_2_O_4_ nanoparticles prepared at various urea molar concentrations. The presence of cobalt, manganese, and oxygen is indicated by prominent peaks in all EDS spectra. All spectra reveal MnCo_2_O_4_ nanoparticle stoichiometry, which is well-matched with XRD data, indicating the phase development of the MnCo_2_O_4_ nanoparticles. In addition, transmission electron microscopy (TEM) was employed to look into the surface microstructure of MnCo_2_O_4_ nanoparticles synthesized at 0.3 M urea concentration. Figure 5 shows a TEM micrograph of the MCO-0.3 MU nanoparticle at various magnifications. Figure 5a–c demonstrates a TEM micrograph at magnifications, demonstrating the clearly visible growth of the flower-like structure by the petal structure resembling a wire-like microstructure. All of the nanowires are clumped together, producing a petal-like shape with some open space in between. Each nanowire is made up of small nanoparticles growing vertically to form a wire-like structure, as seen in the Figure 5c. These wires link together laterally, offering a petal-like structure with free spacing between the wire and tiny nanoparticles, which is more advantageous for the extraction of the electrolyte and electrode during charging and discharging via ion insertion and extraction. Perhaps as a result of this space, the electrode produced with MCO-0.3 MU nanoparticles exhibits remarkable electrochemical performance and the highest energy storage capacity. Figure 5d also depicts the MCO-0.3 MU nanoparticle’s selected area electron diffraction (SAED) pattern, which demonstrates a ring-like pattern, showing that the nanoparticles are polycrystalline in nature. The SAED results confirm the XRD outcomes, pointing to the polycrystalline nature of MnCo_2_O_4_ nanoparticles. EDS analysis was used for further study of the constituent elements of the MCO-0.3 MU nanoparticle. The EDS spectra of MCO-0.3 MU nanoparticles appear in Figure 5e, illustrating the existence of cobalt, manganese, and oxygen. The EDS data correlate well with the XRD stoichiometry and phase of the nanoparticle.

### 3.3. Electrochemical Performance

**Figure 6:**
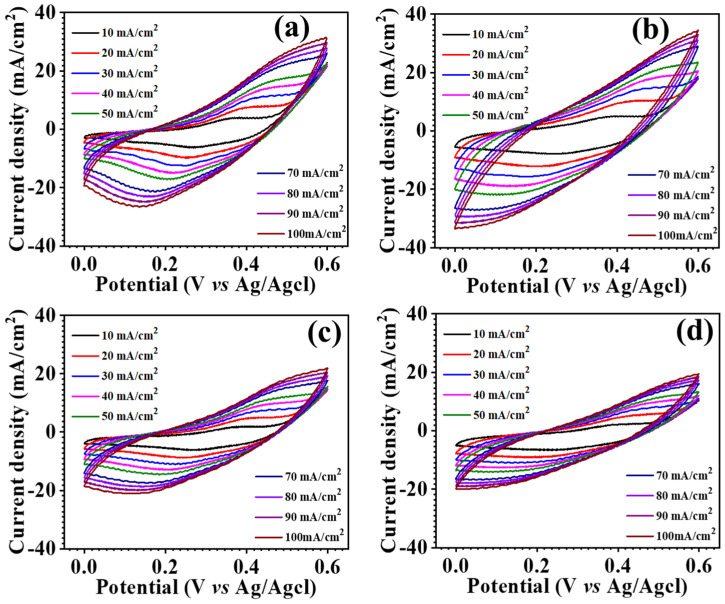
Cyclic voltammetry (CV) profile of MnCo_2_O_4_ nanoparticles, MCO-0.2 MU (**a**), MCO-0.3 MU (**b**), MCO-0.4 MU (**c**), MCO-0.5 MU (**d**).

**Figure 7:**
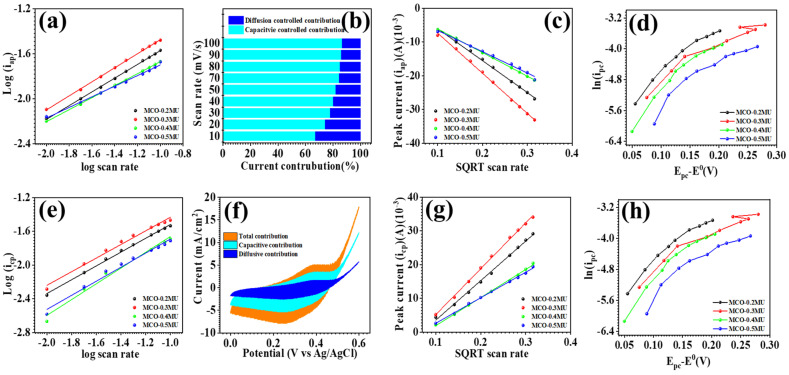
Log *i_p_* vs. log *v* (**a**,**e**) of MnCo_2_O_4_ electrodes prepared at different molar concentrations of urea, current contribution at different scan rates (**b**), current contribution at 10 mV/s (**f**) of MCO-0.3 MU electrode, *i_p_* vs. *v*^0.5^ (**c**,**g**) and ln(*i_p_*) vs. E_p_-E^0^ (**d**,**h**) of MnCo_2_O_4_ electrodes prepared at different molar concentrations of urea.

The synthesized MnCo_2_O_4_ nanoparticles were electrochemically studied using cyclic voltammetry (CV), galvanostatic charge-discharge (GCD), and electrochemical impedance spectroscopy (EIS). The electrochemical measurements have been carried out using aqueous 2 M KOH electrolyte. Figure 6 examines the comparative CV profiles of all electrodes in the potential range of 0.0–0.6 V. The findings show that the urea concentration substantially influences the electrode’s electrochemical performance. The MCO-0.3 MU electrode has a greater area behind the CV profile than the other electrodes shown in the comparative CV profile depicted in as detailed in coming section. The surface architecture of the MCO-0.3 MU nanoparticles could be contributing to the larger area under the curve. The FESEM details suggest a flower-like form of petals with a nanowire structure and microscopic nanowires made up of tiny nanoparticles, as found in TEM examination. During the charging and discharging processes, these small nanoparticles provide accessible surface space for electrode-electrolyte interaction. The floral structure, with nanowire shape and empty spaces, benefits ion interaction, resulting in a greater area under the CV profile for the MCO-0.3 MU electrode when compared to the other electrodes. Figure 6a demonstrating CV profiles at various scan rates shows that the area beneath the CV profile increases as the scan rate increases, demonstrating that the electrode is reversible. With expanding scan rates, the oxidation and reduction peaks shift away from the formal potential, resulting in a drop in peak intensity. The CV profile also shows that the area under the CV profile for MCO-0.3 MU is bigger than the area under the CV profile for the other electrodes. In addition, the kinetics of the electrode was examined by looking at the *b* values. The *b* value is the slope of the $\log\left(i_{p}\right)$ vs. log(v), as calculated from Equation (1) and Figure 7a,e for all electrode materials. According to the *b* value analysis, the charge storage mechanism is either diffusive (0.5) or capacitive (1.0) [31,32,33]. The computed *b* values for all electrodes fall between 0.47 and 0.62, suggesting a combination of both processes.

(1) $$\log\left(i_{p}\right)=\log\left(a\right)+b\cdot log(v)$$

(2) $$i_{p}=k_{c}v+k_{d}v^{1/2}$$

To determine the capacitive and diffusive current contributions, we evaluated the CV profiles at a constant potential and used Equation (2). The slope of the equation indicates the capacitive controlled contribution, while the intercept indicates the contribution controlled diffusion [34,35,36,37,38]. The MCO-0.3 MU electrode’s b value (0.62) signifies contribution from both capacitive and diffusive processes. Figure 7b depict capacitive and diffusion current contributions at different scan rates, with a single Figure 7f demonstrating these contributions for the MCO-0.3 MU electrode at 100 mV/s. The surface microstructure of MnCo_2_O_4_ nanoparticles, which exhibits a flower-like morphology with petal structures made up of tiny particles arranged in nanowires, is attributed to the dominance of diffusive current contribution over capacitive current contribution for MCO-0.3 MU. This observation is consistent with the diffusion coefficient calculations that were calculated from Equation (3) [39,40], which show that the MCO-0.3 MU electrode has higher values for oxidation and reduction (4.31 × 10^−8^ and 5.9 × 10^−8^ cm^2^/S) than other electrodes (values listed in Table 1).

(3) ip=0.4463ACFnnFvDRT

(4) $$i_{p}=0.227nCAFk^{0}exp\left(-\left(\frac{\propto nF}{RT}\right)(E_{p}-E^{0})\right)$$

The electrochemical reaction process has been defined as quasireversible, reversible, or irreversible depending on the K^0^ values that were calculated from Equation (4) [31,41,42]. The MCO-0.3 MU electrode’s k^0^ value (10.1 × 10^−5^ and 6.64 × 10^−5^ cm/s) for charging and discharging suggests a quasireversible electrochemical reaction. The transfer coefficient, which represents the reaction process, is between 0 and 1 and was calculated using Equation (4) [43]. The difference in k^0^ and transfer coefficient can be attributed to varying urea concentrations modifying the surface microstructure. As supported by the b value, higher diffusion coefficient, and dominance of diffusion current contribution at constant potential, a concentration of 0.3 M urea appears to be the optimized molar concentration, providing a large area under the CV profile and active sites for electrode-electrolyte interaction. 

**Figure 8:**
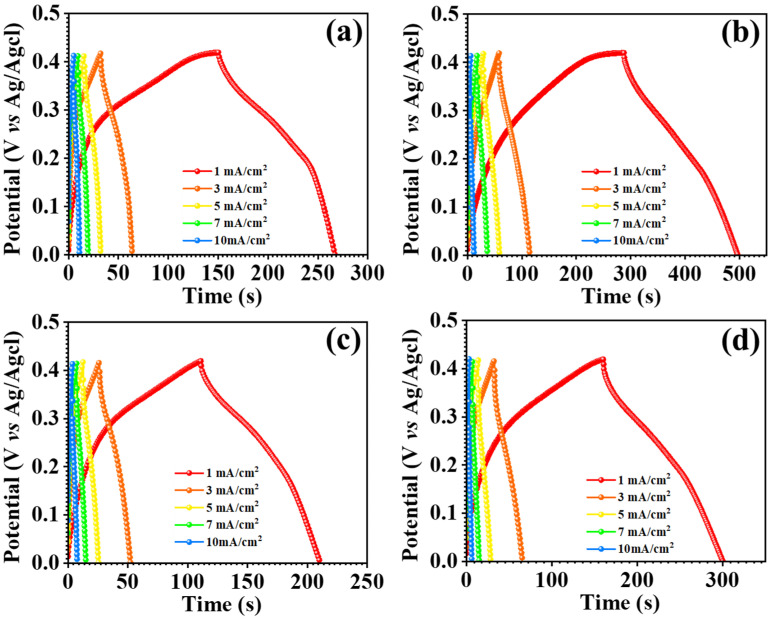
Galvanostatic charge discharge (GCD) profile of MnCo_2_O_4_ nanoparticles, MCO-0.2MU (**a**), MCO-0.3MU (**b**), MCO-0.4MU (**c**), MCO-0.5MU (**d**).

**Figure 9:**
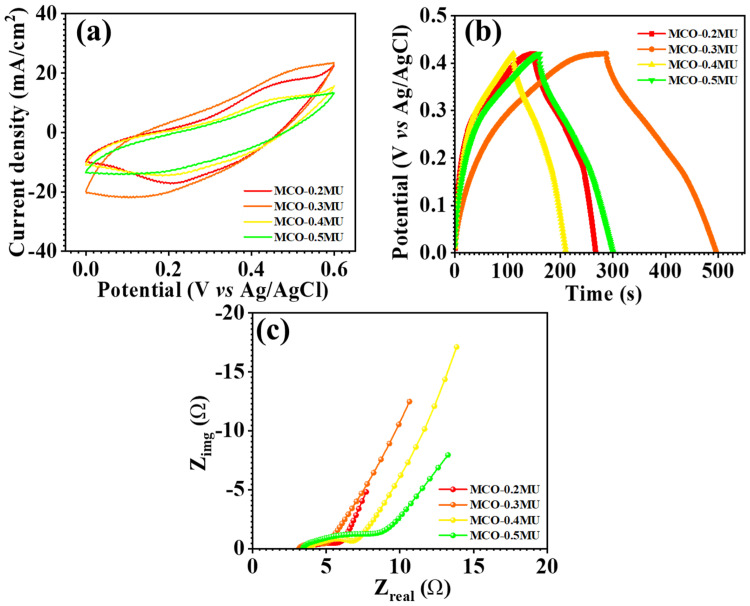
Comparative cyclic voltammetry CV (**a**), galvanostatic charge discharge GCD (**b**) and EIS (**c**) profile of MnCo_2_O_4_ nanoparticles prepared at different molar concentrations of urea.

The GCD measurements for each nanostructure were utilized to look into the electrochemical performance of the electrode material, incorporating specific capacitance, energy density, and power density, as well as the asymmetric supercapacitor device. Figure 9b illustrates the GCD profiles of all electrodes in comparison, with the MCO-0.3 MU electrode having the largest charging and discharging intervals and GCD profile of all electrode, as shown in Figure 8a–d. The effect of raising the urea concentration on surface microstructures is shown in the GCE profiles. The surface microstructure of the MCO-0.2MU electrode was flower-like, with a compact petal-like shape. Its effect on electrode charging and discharging may be attributed to the limited area available for electrolyte-electrode interaction as a result of the compactness of the petal shape. Once the molecular concentration was tuned to 0.3 M, the flower’s surface with free petals demonstrated favorable charging and discharging times. This might be because the free petals impart sufficient space for ion intercalations and deintercalation thus offering few challenges during the charging and discharging processes. By interconnecting the petals of the flower structures laterally with a urea concentration of 0.4 M, the surface morphology converted into a sheet-like structure. Because of the compactness of the sheet shape, this transition resulted in shorter charging and discharging times. Similarly, at 0.5 M urea concentration, a minor overgrowth of MnCo_2_O_4_ nanoparticles on the sheet surface was detected, resulting in a modest increase in charging and discharging periods. The electrodes’ specific capacitance, energy density, and power density have been determined using the Formulas (5)–(7) [44,45,46] and are reported in Table 1.

(5) $$C_{s}=\frac{I_{d}{\cdot T}_{d}}{A\cdot dV}$$

(6) $${ED}_{s}=\frac{{C_{s}\cdot V}^{2}}{7.2}$$

(7) $${PD}_{s}=\frac{3600\cdot {ED}_{s}}{T_{d}}$$

The MCO-0.3MU electrode has a high energy storage capacity of 342 F g^−1^ at a current density of 1 mA/cm^2^ and an energy density of 8.4 Wh/kg at a power density of 140 W/kg. These remarkable MCO-0.3MU electrode findings were used to investigate the asymmetric supercapacitor using activated carbon as the other electrode and a 2 M KOH aqueous electrolyte. Electrochemical impedance spectroscopy (EIS) measurements were used to investigate the electrode transfer nature of all electrodes. Figure 9c depicts the MnCo_2_O_4_ electrode’s Nyquist plot, where the straight line in lower frequency areas shows electrolyte diffusion. The intercept on the x-axis indicates the series resistance (R_s_) at higher frequency areas, which occurs owing to the resistance of the electrode material, electrolyte, and electrode-electrolyte interface [46,47,48,49,50]. The charge transfer resistance (R_ct_) is represented by the semicircle diameter in EIS. Table 2 shows the R_s_ and R_ct_ values for all electrodes. R_s_ and R_ct_ values for the MCO-0.3 MU electrode were 3.11 Ω and 1.76 Ω, respectively. Smaller R_s_ and R_ct_ values might be attributed to the surface microstructure of the MnCo_2_O_4_ electrode. The flower-like surface shape with free-space petals creates an obstacle-free surface, allowing for great energy storage capacity via simple electrode-electrolyte interaction. 

### 3.4. Device Electrochemical Study

**Figure 10:**
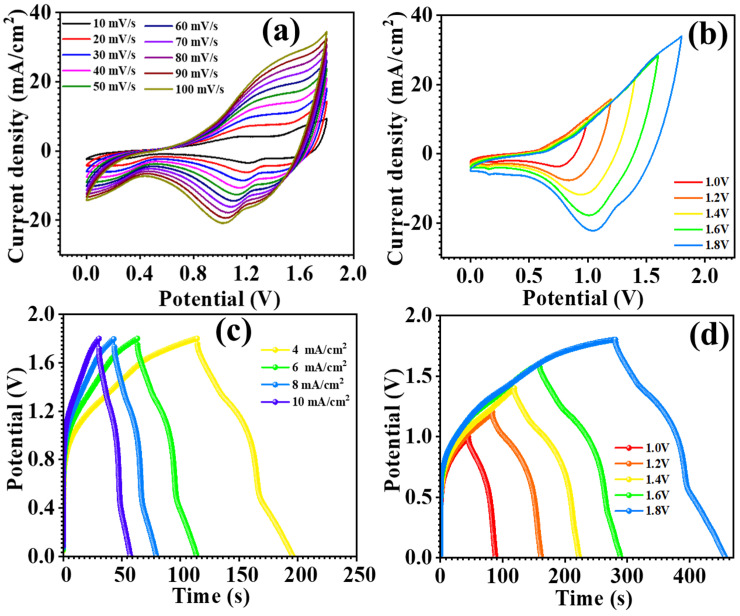
CV profile at different scan rates (**a**), CV profile at potential (**b**), GCD profile at different current densities (**c**), GCD profile at potential (**d**) of asymmetric supercapacitor (ASC).

**Figure 11:**
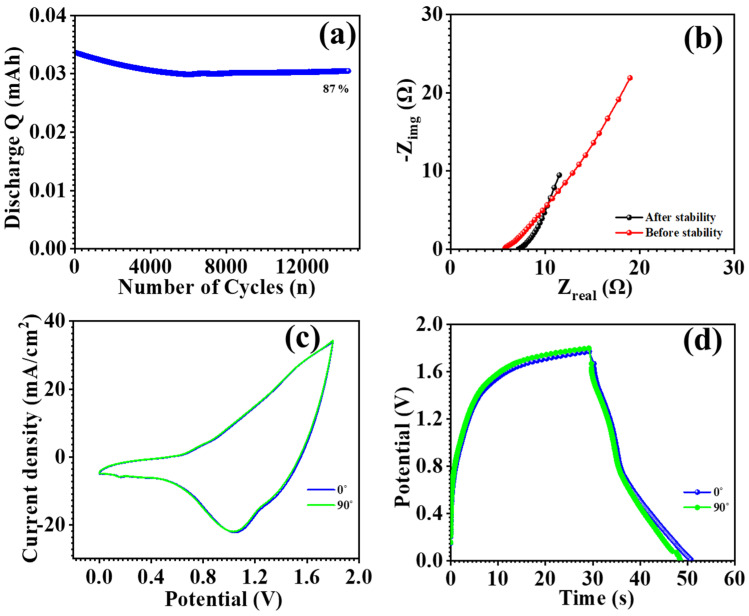
GCD cyclic stability (**a**), EIS spectra (**b**) before and after cyclic stability, CV profile at 0° and 90° (**c**), GCD profile at 0° and 90°, (**d**) of asymmetric supercapacitor (ASC).

The optimized electrode’s practical application was studied through the fabrication of an asymmetric supercapacitor device by employing MnCo_2_O_4_ (MCO) nanoparticles at 0.3 M urea concentration and an activated carbon (AC) electrode. The AC electrode and the MCO-0.3 MU electrode were constructed in an approach exactly as described in the electrode preparation section. The AC electrode and MCO-0.3 MU electrode were placed in a device, with filter paper acting as a separator between the two. A 2 M KOH electrolyte was used, and the device was firmly covered in paraffin paper to prevent electrolyte loss. The potential window of the MCO-0.3 MU//AC asymmetric supercapacitor (ASC) device was determined using cyclic voltammetry (CV) profiles. CV profiles of the MCO-0.3 MU//AC ASC device were acquired throughout several potential windows ranging from 1.0 V to 1.8 V. Up to 1.8 V, the potential rose steadily with the current, as seen in Figure 10b. The potential window of 1.8 V was set, and CV profiles were measured at various scan speeds (10–100 mV/s) as shown in Figure 10a. As the scan rate increased, so did the area beneath the CV profile. Figure 10d shows the galvanostatic charge-discharge (GCD) profiles of the MCO-0.3 MU//AC ASC device measured at various potential windows to confirm its performance. Using a potential window of 1.8 V, GCD profiles were detected at scan speeds ranging from 4–10 mA/cm^2^ as shown in Figure 10c. Equations (5)–(7) were used to determine the specific capacitance, energy density, and power density of the MCO-0.3 MU//AC ASC device. The device has a specific capacitance of 29 F g^−1^ at 4 mA/cm^2^, energy density of 13 Wh/kg, and power density of 553 W/kg. Figure 10a shows the stability of MCO-0.3 MU//AC ASC for practical applications, and the MCO-0.3 MU//AC ASC device maintained 87% of its initial performance after 14,000 cycles. The interaction between electrodes and electrolytes was investigated using electrochemical impedance spectroscopy (EIS). Figure 10b shows EIS spectra of the MCO-0.3 MU//AC ASC device before and after stability testing revealed a rise in series resistance (R_s_) from 5.52 Ω to 6.91 Ω. The minor increase in series resistance might be attributable to surface alterations during lengthy stability testing. The mechanical flexibility of the ASC was tested by bending of the flexible ASC at 90°. Figure 11c,d shows the CV and GCD profile of the ASC at 0° and 90°. CV and GCD profiles show similar profiles and were overlapped with each other showing excellent flexibility. Overall, the study shows that electrodes made with a 0.3 M urea concentration have outstanding electrochemical performance, which is due to the distinctive surface shape of MnCo_2_O_4_ nanoparticles. The ASC device made with MCO-0.3 MU and AC, has a remarkable energy density and power density, indicating that MnCo_2_O_4_ nanoparticles synthesized at 0.3 M concentration have the potential to be useful electrodes for energy storage in supercapacitor applications.

### 3.5. HER and OER Electrocatalysis Study

**Figure 12:**
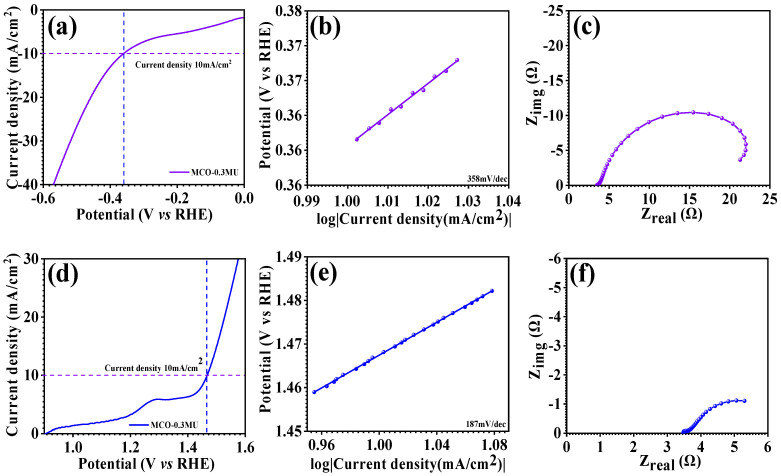
LSV profile (**a**), Tafel plot (**b**) and EIS spectra (**c**) of MnCo_2_O_4_ (MCO-0.3 MU) electrode for HER, LSV profile (**d**), Tafel plot (**e**) and EIS spectra (**f**) of MnCo_2_O_4_ (MCO-0.3 MU) electrode for OER.

In order to study the electrocatalytic activity of the improved MCO-0.3 MU electrode, we used linear sweep voltammetry to measure the HER and OER. At a scan rate of 5 mV/s, the HER and OER LSV were operated in an aqueous electrolyte containing 1 M KOH. The three electrodes utilized in this experiment: a working electrode made of MCO-0.3 MU, a platinum electrode which served as counter electrode, and an Ag/AgCl electrode which served as a reference electrode. The potentials were converted into RHE from equation [51,52,53]. Figure 12a,d shows LSV plots for HER and OER, respectively. In comparison to RHE, the MCO-0.3 MU electrode displays overpotential of 358 mV and 1.46 V, respecctively. The Tafel slopes were obtained from the equation seen in Figure 12b,e. The MCO-0.3 MU electrode’s Tafel slope for HER is 356 mV/dec and for OER it was 187 mV/dec. Figure 10c,f show the EIS spectra of MCO-0.3 MU for HER and OER, through EIS analysis recorded in a 1 M KOH electrolyte at a scan rate of 5 mV/s over a frequency range of 0.1 Hz to 100 kHz. The electrocatalytic processes of HER and OER were investigated. For HER, the electrode’s R_s_ value is 3.31 Ω, whereas for OER, it is 3.43 Ω. The nanowire in the floral surface shape of the MnCo_2_O_4_ nanoparticle could be responsible for the remarkable overpotential and Tafel slope of the MCO-0.3 MU electrode for HER and OER. Extra electrolytic surface area, provided by access to the flower’s available free space, demonstrates exceptional electrocatalytic activity. These outcomes indicate the potential that MCO-0.3 MU might be an attractive electrode material for water electrocatalysis.

## 4. Conclusions

Varying the molar concentration of urea effectively changed the surface microstructure of MnCo_2_O_4_ nanoparticles using a hydrothermal method. The surface modifications significantly affected the electrode’s chemical performance. In a three-electrode arrangement, the MnCo_2_O_4_ nanoparticles prepared at a 0.3 M urea concentration displayed a flower-like microstructure. Furthermore, the MnCo_2_O_4_ electrode exhibited a superior specific capacitance of 342 F g^−1^ and an energy density of 8.4 Wh/kg at a power density of 140 W/kg. Studies into electrochemical impedance revealed a charge transfer resistance of 1.79 Ω and a series resistance of 3.11 Ω. MnCo_2_O_4_ nanoparticles and activated carbon electrodes were used to develop asymmetric supercapacitors (ASCs). The ASCs demonstrated a remarkable 29 F g^−1^ specific capacitance at 4 mA/cm^2^ current density and 13 Wh/kg energy density at 553 W/kg power density. According to cyclic stability testing, the ASCs retained 87% of their capacity across 14K GCD cycles. EIS measurements of the ASCs found a series resistance of 5.54 Ω before cyclic stability and 7.01 Ω after cyclic stability. Electrocatalysis investigations revealed overpotential of 358 mV and 1.48 V for HER and OER, respectively, with Tafel slopes of 356 mV/dec and 187 mV/dec. Series resistances for both the HER and OER from EIS experiments were 3.48 Ω. Together with the stability of ASC devices, the remarkable specific capacitance, energy density, and power density of ASCs as well as the exceptional electrocatalytic HER and OER performance of MnCo_2_O_4_ nanoparticles show potential for a wide range of applications.

## Figures and Tables

**Table 1:** Comparison of diffusion coefficient, C_s_, ED and PD MnCo_2_O_4_ electrodes prepared at different molar concentrations of urea.

Sample Code	Diffusion Coefficient (cm^2^/S) 10^−8^	Specific Capacitance (F g^−1^)
MCO-0.2 MU	3.07	251
MCO-0.3 MU	4.21	342
MCO-0.4 MU	1.71	161
MCO-0.5 MU	1.38	220

**Table 2:** Comparison of standard rate constant (k^0^), transfer coefficient (α), series resistance (R_s_), ED and charge transfer resistance (R_ct_) MnCo_2_O_4_ electrode prepared at different molar concentration of urea.

Sample Code	Standard Rate Constant k^0^ (cm/S) 10^−5^	Transfer Coefficient (α)	Series Resistance (R_s_) (Ω)	Charge Transfer Resistance (R_ct_) (Ω)
MCO-0.2 MU	5.18	0.32	3.61	2.24
MCO-0.3 MU	6.64	0.22	3.11	1.76
MCO-0.4 MU	2.71	0.35	3.83	2.95
MCO-0.5 MU	3.01	0.26	3.35	4.89

## Data Availability

Data are contained within the article.
